# Factors Affecting Patient Safety Culture from Nurses’ Perspectives for Sustainable Nursing Practice

**DOI:** 10.3390/healthcare10101889

**Published:** 2022-09-28

**Authors:** Sally Mohammed Farghaly Abdelaliem, Samira Ahmed Alsenany

**Affiliations:** 1 Department of Nursing Management and Education, College of Nursing, Princess Nourah bint Abdulrahman University, P.O. Box 84428, Riyadh 11671, Saudi Arabia; 2 Department of Community Health Nursing, College of Nursing, Princess Nourah bint Abdulrahman University, P.O. Box 84428, Riyadh 11671, Saudi Arabia

**Keywords:** patient, safety culture, nurses, perspectives, sustainable nursing practices

## Abstract

Individual and group beliefs, attitudes, perceptions, competences, and behavioral patterns all contribute to the safety culture of a healthcare company. The study’s goal is to assess nurses’ perceptions of elements that influence patient safety culture in order to promote long-term nursing practice. A descriptive cross-sectional study design was done among a sample of 146 nurses who were recruited from one hospital in Egypt. They completed a self-administered, printed questionnaire. The questionnaire assessed participants’ socio-demographic data and their perception regarding patient safety culture for sustainable nursing practices. The findings revealed that nursing staff had a high perception regarding patient safety culture a with mean score (159.94 ± 7.864). Also, the highest percentage (74.66%) of had no safety events reported yearly. Creating a unit-specific patient safety culture suited to the competences of the unit’s RNs in patient safety practice would be crucial to increasing and sustaining high levels of patient safety attitudes, skills, and knowledge among the unit’s RNs, influencing patient safety. When implementing interventions to promote patient safety and reporting culture in hospitals, policymakers, hospital administrators, and nurse executives should take the current findings into account. A multidimensional network intervention addressing many elements of patient safety culture and integrating different organizational levels should be implemented to enhance patient safety and a no-blame culture.

## 1. Introduction

Individual and group beliefs, attitudes, perceptions, competences, and behavioral patterns all contribute to the safety culture of a healthcare company. These actions define an organization’s commitment to the style and competency of health and safety management [1]. Healthcare organizations with a positive safety culture are distinguished by mutual trust-based communication, shared perceptions of the importance of safety, trust in the efficacy of preventive measures, positive beliefs about how things work in the organization, and the interaction of these with work unit and organizational structures, as well as systems, which produce behavioral norms in healthcare organizations that promote patient safety [2].

The patient safety phenomenon emerged in the 1990s, sparking interest in the evaluation and influence of organizational features on patient safety. According to the assessment results, the specified aspects inside an organization that influence the day-to-day operation of the company include organizational culture, communication, feedback systems, and leadership style [3]. Because patient safety is a continuous worry within the healthcare profession and continues to be a concern for the leaders of health care organizations, it is critical that an organization offers the safest treatment possible to patients. As a result, the emphasis is on providing safe patient care on a daily basis in order to grow into an organization that values patient safety [4].

Because patient safety is a fundamental human right, it is the responsibility of all healthcare professionals, regulatory agencies, and political organizations to foster a growing patient safety culture [5]. According to Boamah et al. (2018) [6], “leaders have the power to influence nurses’ safety behaviors by encouraging subordinates to be actively involved in safety practice measures and to be compliant with the safety process.” According to the World Health Organization (WHO), “the main barrier for moving toward a safer health system is shifting the culture from one of blaming people for errors to one in which errors are seen as chances to improve the system and prevent injury”. As a result, healthcare organizations should foster an atmosphere in which patient safety culture is explicitly stated as an organizational goal and is prioritized by healthcare executives in order to promote patient safety culture (WHO, 2017) [7].

Patient safety culture is a comprehensive notion that includes the nurse’s interactions with healthcare organizational leaders “beginning with hospital managers/administrators, directors of nursing services, or all management roles, peers, professional colleagues, and patients” [8]. Nurses are in charge of ensuring that hospitalized patients get safe treatment, and nurse leaders are in charge of creating priorities that define the safety culture and prevent adverse occurrences [9]. Leadership commitment to patient safety culture has been identified as one of the top features of consistently safe businesses, and they also have the ability to influence patient safety culture more than practicing nurses at the bedside [8].

Creating a safety culture in healthcare organizations necessitates the involvement of all members, as healthcare delivery necessitates multiple caregivers “beginning with physicians, pharmacists, nurses, technicians, and housekeepers” to collaborate as an effective team with the goal of achieving desired patient wishes, outcomes, and preventing harm [10]. Because the quality of cooperation influences the efficacy of treatment, patient safety, and clinical results, the objective of developing a safety culture must be shared by all personnel in order to provide safe and effective healthcare [11].

Sorra and Nieva (2004) [12] described patient safety culture as healthcare organizations’ common philosophies, ideologies, beliefs, sentiments, assumptions, expectations, attitudes, conventions, and values. They classified patient safety culture into three major dimensions: unit level safety culture, hospital wide survey culture, and outcome measures, with twelve sub-dimensions, namely teamwork within units, supervisor/manager expectations and actions promoting patient safety, management support for patient safety, non-punitive response to errors, organizational learning-continuous improvement, staffing, communication openness, feedback and communication about error, teamwork, and teamwork.

The World Health Organization [7] recognized these elements in its study reports and found that the combined findings from these studies ranked medical mistakes as one of the top five major causes of mortality and found that it had an economic impact on healthcare institutions. They also determined that nurses’ views of patient safety are stronger indicators of overall safety culture, and that by concentrating on these results to reduce adverse occurrences, hospital administrators and nurse executives might enhance patient safety. The researchers hope that assessing patient safety culture among nurses working in a university hospital with a high patient capacity and a diverse nursing staff mix will aid in the study of nurses’ perceptions of the factors influencing patient safety culture, which are central to current health-care reform. The assessment of these characteristics, as well as the statistical results of their impact on patient safety culture, will aid healthcare administrators in making scientific and statistically sound decisions about the present healthcare reform process.

### 1.1. Aim of the Study

To evaluate nurses’ perception toward factors affecting patient safety culture for sustainable nursing practice.

### 1.2. Research Question

What is the level of nurses’ perception regarding patient safety culture for sustainable nursing practices?

## 2. Materials and Methods

### 2.1. Research Design and Sampling

This is descriptive cross-sectional research that was carried out in an Egyptian university hospital. To examine their view of patient safety culture, a convenience sample of staff nurses (N = 146) who were available at the time of data collection were included. The nurses’ sample size was calculated using Epi-info 7 with a 5% variance, 95% confidence, and 0.80 power at a 0.5 significance level. Newly employed nurses with fewer than six months of professional experience were excluded.

### 2.2. Measurement

#### Hospital Survey on Patient Safety Culture (HSPSC) Instrument

The survey was created by Sorra and Nieva (2004) [12] and verified by the Agency of Health Care Research and Quality in 2016 [13] to measure nurses’ perceptions of the hospital’s patient safety culture. It comprises 42 questions separated into twelve subdimensions and aggregated into three dimensions: unit safety culture level, hospital-wide safety culture, and outcome measures. The unit safety culture level dimension includes eight sub-dimensions: teamwork within units (four items), supervisor/manager expectations and actions promoting patient safety (four items), organizational learning-continuous improvement (three items), management support for patient safety (three items), non-punitive response to errors (three items), staffing (four items), communication openness (three items), feedback and communication about errors (three items). With regard to hospital-wide safety culture, the dimension comprises two sub-dimensions: cross-unit collaboration (four items), and hospital handoffs and transitions (four items). For outcome measures, this dimension has two subdimensions: overall perceptions of patient safety (four items) and frequency of reported occurrences (three items). Collective answers were given on a five-point Likert scale, based on the questions addressed; the scale ranges from 1 (strongly disagree) to 5 (strongly agree) (strongly agree). It was used to assess the following sub-dimensions: supervisor/manager safety expectations and actions, organizational learning-continuous improvement, teamwork within hospital units, non-punitive error response, staffing, hospital management support for patient safety, hospital handoffs and transitions, teamwork across hospital units, and overall perception of safety.

The following sub-dimensions were measured on a scale of 1 (never) to 5 (always): communication openness, feedback and communication regarding error, and frequency of event reporting. Furthermore, there were two single items that did not have an alpha statistic reported: patient safety grade, which was measured by study subject responses to a scale ranging from (excellent to failing), and responses to the number of events reported in the previous 12 months in the patients’ unit, which ranged from (no event reports to 21 event reports or more). For the 18 negatively phrased items, the score was reversed.

The total scores for all previously mentioned sub-dimensions ranged from 42 to 210, with 42 indicating a low level of nurses’ perception of patient safety culture, 98 indicating a moderate level of nurses’ perception of patient safety culture, and 155 indicating a high level of nurses’ perception of patient safety culture. In addition, the researcher created a nurses’ socio demographic sheet to gather data on nurses, which includes age, gender, working unit, educational level, position, years of experience in the hospital, and years of experience in the present working unit.

### 2.3. Validity and Reliability

Cronbach’s alpha coefficient test was used to assess the instrument’s reliability by measuring the internal consistency of items. Where Cronbach’s alpha = (0.842) for HSPSC, the instrument validated dependability. In addition, pilot research on 10% of nurses was conducted to validate the instrument’s validity and reliability. Cronbach’s alpha for the dimensions and subdimensions of the HSPSC was clarified as follows: unit safety culture level, hospital-wide safety culture, and outcome measures. With regard to unit safety culture level (α = 0.780), this dimension includes eight sub-dimensions: teamwork within Units (four items, α = 0.823), supervisor/manager expectations and actions promoting patient safety (four items, α = 0.778), organizational learning-continuous improvement (three items, α = 0.861), management support for patient safety (three items, α = 0.794), non-punitive response to errors (3 items, α = 0.760), staffing (4 items, α = 0.772), communication openness (3 items, α = 0.770), feedback and communication about error (3 items, α = 0.862). Hospital-wide Safety Culture (α = 0.897): This dimension comprises two sub-dimensions: cross-unit collaboration (four items, α = 0.854), and hospital handoffs and transitions (four items, α = 0.876). The dimension of outcome measures has two subdimensions: overall perceptions of patient safety (four items, α = 0.725) and frequency of reported occurrences (three items, α = 0.734).

### 2.4. Data Collection

The hospital’s administration gave written permission for data collecting. Nurses were given a printed research questionnaire. To assess the feasibility of the investigation, 10% of the study sample (*n* = 14) participated in a pilot study; individuals in the pilot study were eliminated from the study sample. The pilot research revealed that the study instrument was clear and that no changes were required. The questionnaire took between 10–15 min to complete. Data was collected during a two-month period, from January to March 2021.

### 2.5. Ethical Considerations

Official authorization to gather the needed data was received from the Alexandria University Research Ethics Committee (N 23-12-2021) and the directors of the study settings. The confidentiality and privacy of the data were preserved. The nurses’ signed assent to participate in the study prior to data collection was safeguarded. The participants were given the option to withdraw from the study at any time.

### 2.6. Data Analysis

The data were entered into IBM SPSS version 23’s social sciences statistical tool. Demographic characteristics were presented using frequencies and percentages, whereas continuous variables were presented using mean and standard deviation (SD). All statistical analyses were run with an alpha of 0.05.

## 3. Results

Table 1 showed that the highest percentage of nurses (56.16%) were in the age group of 20 < 30 with a mean score of (23.99 ± 1.542), while the lowest percentage (10.27%) of nurses were in the age group ≥ 50. Concerning nurses’ gender; it was found that the highest percentage was from female nurses (67.12%), while the lowest percentage were male nurses (32.88%). Regarding nurses’ marital status; it was found that the highest percentage (60.27%) of nurses were married, while the lowest percentage (39.73%) were single nurses. Concerning nurses’ working units; it was found that the highest percentage of nurses (23.97%) were working in Medical Units, while the lowest percentage of nurses (5.48%) were working in Recovery Units. Regarding nurses’ educational level; it was found that the highest percentage of nurses (51.37%) had a diploma from a technical institute, while the lowest percentage of nurses (48.63%) had a Bachelor of Science in Nursing. In relation to the nursing position; it was found that the highest percentage of nurses (88.36%) were nurses, while, the lowest percentage of the study subjects (11.64%) were first line nurse managers. Concerning nurses’ years of experience in nursing, it was found that the highest percentage of nurses (100%) with mean score (3.42 ± 1.0829) had experience < 10 years. In relation to nurses’ experience in the current working unit; it was found that the highest percentage of nurses (100%) with mean score (2.97 ± 0.791). 

Table 2 shows that the majority of nurses (88.36%) had a high perception regarding patient safety culture, with a mean score (159.94 ± 7.864). In relation to unit safety culture, it was noted that nurses’ perception regarding "unit safety culture" got a mean score 100.80 ± 4.852, and the sub-dimension “teamwork within hospital units” had the highest mean score (17.88 ± 1.783), while, the sub-dimension “staffing” had the lowest mean score (8.29 ± 1.467). Concerning hospital-wide safety culture, it was found that this dimension had a mean score of 32.42 + 2.739, and the sub-dimension “hospital handoffs and transition” had the highest mean score (17.75 ± 2.009) and the sub-dimension “teamwork across hospital units” had the lowest mean score (14.66 ± 1.250). Regarding the outcome measures dimension, it was found the “outcome measures” dimension had the lowest mean score (26.72 + 2.556), as the sub-dimension “overall perception of patient safety” had the highest mean score (16.27 ± 1.152) than the sub-dimension frequency of event reporting which had the lowest mean score (10.45 ± 2.222) (Table 3).

Table 4 revealed that the highest percentage of nurses (55.48%) had a very good perception regarding overall grade of patient safety culture with a mean score of 4.35 + 0.606. Table 5 revealed that the highest percentage (74.66%) of nurses’ perception toward number of events reported yearly had no events reported, while the lowest percentage was (1.37%) of nurses who reported (three to five) events yearly.

## 4. Discussion

Healthcare companies’ safety culture serves as a guide for healthcare personnel to know how to behave in the workplace and what behaviors are appropriate for overcoming patient safety issues that affect patient outcomes [14]. Building a patient safety culture requires healthcare organizations to define their current safety culture, use teamwork and communication on a daily basis and in operations, understand what the leadership role is, investigate how to maintain the safety culture once established, recognize that there will be barriers along the way, and look at best practice organizations that focus on patient safety for a guide to success and profitability [15].

The current study findings revealed that nurses had a positive perception of patient safety culture, which could be attributed to ongoing education and training on safety measures for nurses as a requirement for in-service training, such as infection control training measures, cardio-pulmonary resuscitation, effective communication through the staff development department, and supported collaboration, cooperation, and coordination of nurses within and across hospitals. These findings are reinforced by Amiri et al. (2018) [15]; Aboufour & Subbarayalu, (2022) [16]; and Aljohani & Alsharqi, 2021) [17], who discovered that patient safety culture varies considerably not just between hospitals, but also by clinical standing and employment class within particular institutions. Furthermore, Mears et al. (2019) [18] discovered a favorable association between employee engagement and safety culture among nurses. In contrast, the findings of this study contradicted those of Alquwez et al. (2018) [19] and Hessels et al. (2019) [20], who discovered a negative view of patient safety culture among health care personnel.

Furthermore, the current study’s findings demonstrated that nurses had a positive opinion of patient safety culture at the unit level. This could be attributed to the presence of management support for patient safety, the development of good communication between nurses and between nurses and their managers, as well as feedback about errors, nurses working as a team within hospital units, and continuous learning and improvement through attendance at training programs at both hospitals under study. This conclusion was consistent with and confirmed by Araújo et al. (2022) [14], and Olsen & Leonardsen (2021) [21], who explained that the overall emphasis in quality and safety initiatives appears to be stressing features that change more at the unit level than at the hospital level. Furthermore, nurse leaders’ patient safety actions are related to how nurses evaluate their organizations and unit’s ability to provide safe patient care. These findings, on the other hand, contradicted the findings of Alquwez et al. (2018) [19], who reported that patient safety culture at the unit level received a moderate impression and was identified as a possible development area.

Nurses’ perceptions of patient safety culture at the hospital-wide level dimension revealed that the two sub-dimensions had a high mean score of nurses’ perceptions at the two hospitals regarding teamwork across hospital units and hospital handoffs, and transitions and were regarded as areas of strength. The perception of staff nurses was greatest in the hospital handoffs and transitions subdimension of hospital-wide safety culture. This might be connected to a good interchange of patient information between staff nurses inside the hospital during shift changes or during patient referral to another unit within the hospital, as well as collaboration between teams within and between hospital units. As a result, it was seen as a source of strength. These findings were consistent with those reported by Alsabri et al. (2022) [22] and Wu et al. (2022) [23], who discovered a favorable attitude toward hospital handoffs and transitions among the nurses surveyed. These findings contrasted with those of Alsabri et al. (2022) [22] and Alquwez et al. (2018) [19], who found that hospital handoffs and transitions received a moderate percentage and were regarded as an area for improvement due to the presence of missing information during shift changes and incomplete shift reports among the studied staff. In terms of the outcome measures component of safety culture, there was a strong response from staff nurses to overall perceptions of patient safety and the frequency of incident reporting. These findings were reinforced by Kakemam et al. (2021) [24], who demonstrated that effective and high perceptions of cooperation by staff nurses enhance clinical care process and patient outcome, including harm and adverse events reduction. Mears et al. (2019) discovered that there is no significant association between safety culture and patient outcome, however Alquwez et al. (2018) [19] discovered that the outcome dimension received a low percentage and was deemed a weakness.

Nurses had a very good overall opinion of patient safety culture, which might be attributed to fewer patient complaints documented in their working units and the delivery of safe patient care. The hospital administration offers a good system that prevents errors from occurring, and no severe blunders have occurred or have been recorded. These findings are congruent with those published by Alsabri et al. (2022) [22] and Wu et al. (2022) [23], who found that the examined healthcare organizations had a higher overall sense of patient safety culture. This finding, on the other hand, contrasted with Arajo et al. (2022), and Olsen & Leonardsen (2021) [21], who discovered that the overall perception of patient safety had a moderate percentage and was considered an area for improvement due to hospital management, which values patient safety problems to a certain extent.

In terms of incident reporting frequency, the majority of nurses had no event reports yearly, which is seen as an essential issue for development. This might be due to an unclear communication route, rigorous time pressure for staff and economic reasons such as the ineffective and inefficient utilization of resources such as time, budget, materials and supplies that leads to precarious situations for personnel and sub-optimal care, and staff nurses may be fearful about discussing and reporting problems that may occur at the hospital. These findings were consistent with and supported by the findings of Lee and Dahinten, (2021) [25], who discovered that the frequency of reported occurrences had a modest mean score and was seen as a potential area for development.

In relation to staffing sub-dimensions of unit level safety culture, it had the lowest mean score of nurses’ perception and was considered as an area for improvement. This could be due to the shortage of staff nurses, the overload of work perceived by the respondents and increased patient admission rates with their demand for a high quality of healthcare. This finding was consistent with what was reported by Elmontsri, Almashrafi, and Banarsee, (2017) [26] who reported that the area of staffing needs continuous improvement because the organizations continually need adequate staff to overcome the workload. On the other hand, this finding was contrasted with what is reported by the Agency of Healthcare Research and Quality (2012) [27], which reported that the charge nurses who made mindful staffing decisions were more effective in reducing chaos in patient units to decrease the perception of shortages and work overload.

### 4.1. The Study’s Limitations

The outcomes of this study have significantly added to the previous studies on patient safety culture perception. The study, however, should be viewed in light of its limitations. The subjects were recruited from a specific location for convenience; therefore, the generalizability of the results is limited. Furthermore, because the current results are based on self-reported data, they are vulnerable to response bias and subjectivity. Furthermore, this study just describes the study variables; no contributing relationship can be demonstrated. In the future, longitudinal, experimental, and multi-site (such as among different countries in Africa) research may help to solve these limitations and the results at that time can be representative for other hospitals in Egypt/ Africa if a national study was conducted using the same technique. The work atmosphere in the current study context may have affected patient safety culture to a high degree. This organization has made human resource development a top priority. Nurses have been urged to participate in continuous professional development programs for patient safety.

### 4.2. Relevance to Clinical Practice

This report provides critical information and raises awareness about serious patient safety issues in Egypt. A high perception of patient safety culture among nurses, as well as the absence or reporting of patient safety events, may be due to their level of education, years of service in the hospital, and current work areas or units, as well as staff nurses who do not have direct contact or interaction with patients. Policymakers, hospital administrators, and nurse executives should take the current results into account when designing interventions aimed at sustaining and enhancing the patient safety culture in hospitals, and allow nurses who are process honors to participate in patient safety related issues (as evaluation, planning, budgeting, controlling and risk assessment) and to have a voice in decision making with activation to no blame culture and give them the confidence in their opinion as it may lead to improving the nursing practice to promote patient safety. A multidimensional network intervention addressing the numerous dimensions of patient safety culture and integrating different organizational levels should be carried out to enhance patient safety, taking into consideration the diverse processes in patient safety culture. Hospitals should pay more attention to the problems that have been found. Various transitions of care models, such as the Care Transitions Intervention [28], that address the root causes of ineffective patient care transitions (e.g., communication, patient education, and accountability breakdowns), can be adopted and incorporated into the multidimensional intervention to improve handoffs and transitions.

To promote communication openness, a comprehensive provider or team communication strategy that includes a structured communication tool, a standardized escalation mechanism, daily multidisciplinary patient-centered rounds with a daily objectives sheet, and team huddles can be implemented. The SafetyNET (an organization-wide patient safety network comprised of education, an online interactive discussion forum, and a recognition program or newsletter), which has been shown to improve overall safety event reporting and minimize moderate-adverse classified events, may also be implemented [29]. However, these ideas must be evaluated for their usefulness in the current context. Finally, the need of leadership involvement in developing a robust and long-lasting patient safety culture is emphasized [30]. Furthermore, hospital administrators may improve safety by engaging and interacting with frontline employees on a regular basis through strategic initiatives.

## 5. Conclusions

The current study found that nurses had a good attitude toward patient safety culture, which may have an influence on long-term patient safety practices in the study settings. The need for appropriate interventions to improve and sustain quality improvement approaches, such as staff-to-patient safety culture orientation and the construction of an effective incident reporting system, can have a substantial impact on nurses’ overall patient safety culture evaluations. Although nurses rated their patient safety practices as very good, several aspects of patient safety culture, particularly those related to the establishment of a nonpunitive culture and the teaching of nurses working across all units, regardless of their level of education or years of service, should be improved. The study’s findings are expected to result in the creation of appropriate therapies for long-term patient safety measures in Egyptian hospitals. Furthermore, patient safety culture is a significant issue that every hospital administrator and leader should investigate in order to promote change and deliver a quality and safe hospital environment.

## Figures and Tables

**Table 1:** Socio-Demographic and Work-Related Characteristics of the Study Subjects.

Nurses Socio-Demographic Characteristics	University Hospital (*n* = 146)	
	N	%
Age		
▪ (20 < 30)	82	56.16
▪ (31 < 40)	30	20.55
▪ (41 < 50)	19	13.01
▪ (≥50)	15	10.27
Mean ± SD	23.99 ± 1.542	
Gender		
▪ Male	48	32.88
▪ Female	98	67.12
Marital status		
▪ Married	88	60.27
▪ Single	58	39.73
Working unit		
▪ Medical Units	35	23.97
▪ Surgical Units	33	22.6
▪ Critical Care Units	23	15.75
▪ Operating Rooms	12	8.22
▪ Recovery Units	8	5.48
▪ Emergency Department	20	13.7
▪ Outpatient Units	15	10.27
Educational level		
▪ Diploma of Secondary Technical Nursing School	0	0.00
▪ Diploma of Technical Health Institute	75	51.37
▪ Bachelor of Science in Nursing	71	48.63
Nurses’ Position		
▪ Bed side nurse	129	88.36
▪ First line nurse manager	17	11.64
Years of experience in nursing		
▪ <10 years	146	10.00
Mean ± SD	3.42 ± 1.082	
Years of experience in the current working unit		
▪ <10 years	146	100.00
▪ ≥10 years	0	0.00
Mean ± SD	2.97 ± 0.791	

**Table 2:** Nurses’ Level of Perception Regarding Patient Safety Culture.

Level of Nurses’ Perception Regarding Patient Safety Culture	University Hospital (*n* = 146)	
	N	%
▪ Moderate	17	11.64
▪ High	129	88.36
Mean ± SD	159.94 ± 7.864	

(42–97) Low level, (98–154) Moderate level, (155–210) High level

**Table 3:** Nurses’ Perception Regarding Patient Safety Culture Factors.

Patient Safety Culture Factors	Mean ± SD
	University Hospital
1. Supervisor/manager expectations and actions promoting	16.69 ± 1.783
2. Organizational learning-continuous improvement	12.24 ± 0.956
3. Teamwork within hospital units	17.88 ± 1.783
4. Non-punitive response to error	10.30 ± 2.242
5. Staffing	8.29 ± 1.467
6. Hospital management support for patient safety	11.90 ± 0.905
7. Communication openness	11.48 ± 2.062
8. Feedback and communication about error	12.01 ± 1.180
Total unit safety culture	100.80 + 4.852
9. Teamwork across hospital units	14.66 ± 1.250
10. Hospital handoffs and transitions	17.75 ± 2.009
Total hospital-wide safety culture	32.42 + 2.739
11. Overall perception of safety	16.27 ± 1.152
12. Frequency of event reporting	10.45 ± 2.222
Total outcome measures	26.72 + 2.556

**Table 4:** Nurses’ Evaluation of Overall Patient Safety Culture Grade.

Overall Grade of Patient Safety Culture Grade	University Hospital (*n* = 146)	
	N	%
▪ Poor	2	1.37
▪ Acceptable	4	2.74
▪ Very good	81	55.48
▪ Excellent	59	40.41
Patient safety grade	Mean + SD 4.35 + 0.606	

**Table 5:** Nurses’ Perception Regarding Number of Events Reported.

Number of Events Reported	University Hospital (*n* = 146)	
	N	%
Event reporting		
▪ No event reports yearly	109	74.66
▪ (1–2) event reports yearly	35	23.97
▪ (3–5) event reports yearly	2	1.37
Number of events reported	Mean + SD 1.27 + 0.474	

## Data Availability

The datasets generated and/or analyzed during the current study are not publicly available due to data privacy but are available from the corresponding author on reasonable request.
